# Is Oral Function Associated with the Development of Sarcopenic Obesity and Sarcopenia in Older Adults? A Prospective Cohort Study

**DOI:** 10.3390/diseases13040109

**Published:** 2025-04-05

**Authors:** Sho Shirotori, Yoko Hasegawa, Koutatsu Nagai, Hiroshi Kusunoki, Shogo Yoshimura, Kana Tokumoto, Hirokazu Hattori, Kayoko Tamaki, Kazuhiro Hori, Hiromitsu Kishimoto, Ken Shinmura

**Affiliations:** 1 Department of Comprehensive Prosthodontics, Graduate School of Medical and Dental Sciences, Niigata University, 2-5274 Gakkocho-dori, Chuo-ku, Niigata City 951-8514, Japan; 2 Department of Oral and Maxillofacial Surgery, Hyogo Medical University, Nishinomiya City 663-8501, Japan; 3 Department of Physical Therapy, School of Rehabilitation, Hyogo Medical University, Kobe City 650-8530, Japan; nagai-k@hyo-med.ac.jp; 4 Department of Internal Medicine, Osaka Dental University, Osaka City 540-0008, Japan; 5 Department of General Internal Medicine, Hyogo Medical University, Nishinomiya City 663-8501, Japan; kayoko_tamaki@hotmail.com (K.T.); ke-shimmura@hyo-med.ac.jp (K.S.)

**Keywords:** sarcopenic obesity, obesity, tongue pressure, oral function, hypertension

## Abstract

Background: Sarcopenic obesity, defined as the concurrent loss of muscle mass and adipose tissue accumulation, is associated with reduced physical function and poor health status in older adults. Although oral function can impact the overall health of older adults, its role in the development of sarcopenic obesity remains unclear. Herein, we aimed to examine the association between oral function and the incidence of sarcopenic obesity. Methods: This longitudinal cohort study included 597 independent older adults (aged ≥65 years) from Tamba-Sasayama, a rural region of Japan, who participated in academic studies between June 2016 and December 2023. Participants underwent surveys at least twice, with a minimum two-year interval. The participants were divided into four groups (robust, obese, sarcopenic, and sarcopenic obese) according to their health condition. Sarcopenic obesity was diagnosed based on the guidelines of the Japanese Working Group on Sarcopenic Obesity. The oral function was evaluated by assessing the number of remaining teeth, tongue pressure, occlusal force, masticatory performance, and oral diadochokinesis. Cox proportional hazards regression analysis evaluated the association between oral function and the incidence of sarcopenic obesity after adjusting for relevant confounders. Results: The sarcopenic obesity group was older, had lower skeletal muscle mass, and inferior physical function. This cohort also had the highest prevalence of hypertension and significantly fewer remaining teeth. The proportion of individuals with sarcopenic obesity was 1.7% of the total population, with 2.8% in the obesity group at baseline, and 8.0% of those were diagnosed with sarcopenia progressing to sarcopenic obesity. The Cox regression model revealed that reduced tongue pressure was significantly associated with an increased risk of sarcopenic obesity, with a hazard ratio of 0.906 (95% confidence interval: 0.829–0.990; *p* = 0.028), independent of other variables related to sarcopenia and obesity. Conclusions: Our findings suggest that oral function is associated with the incidence of sarcopenic obesity but not with that of sarcopenia or obesity alone.

## 1. Introduction

Sarcopenia and obesity are two major health concerns impacting the older population and have substantial implications for their overall health and quality of life. Sarcopenia is characterized by the age-related loss of muscle mass, strength, and physical performance [1,2]. In older adults, obesity is associated with an elevated risk of falls, reduced functionality, diminished quality of life, and increased mortality. It also increases the risk of cardiovascular diseases, metabolic disorders, cognitive impairment, and arthritis [3,4].

The interplay between obesity and skeletal muscles in the aging population is complex, with evidence suggesting both protective and deleterious effects. Although obesity is associated with impaired physical function and resistance to anabolic stimuli, it may also lead to greater muscle mass in weight-bearing muscles than in older, lean individuals [5,6].

The coexistence of sarcopenia and obesity leads to sarcopenic obesity, a condition that has garnered increasing attention owing to its profound effects on health outcomes [6,7,8]. Sarcopenic obesity is characterized by muscle weakness due to sarcopenia combined with the metabolic complications of obesity. These conditions exhibit a synergistic relationship, each exacerbating the progression [8]. Sarcopenic obesity is associated with a higher risk of cardiovascular disease, diabetes, and impaired physical function than either condition alone. Sarcopenic obesity involves a vicious cycle of cross-talk between adipose and muscle tissue, and increased white adipose tissue and local muscle fat infiltration leading to inflammatory adipokine secretion, inhibiting protein synthesis and inducing catabolism. Cytokine secretion by fat mass affects muscle tissue and other organs, such as the liver and white adipose tissue, inhibiting insulin signaling and increasing the risk of insulin resistance [9,10]. Additionally, peri-muscular fat plays a critical role in different phenotypes of sarcopenic obesity, influencing inflammatory pathways and metabolic dysfunction. Furthermore, hormone-related responses vary across phenotypes, which may contribute to the secretion of inflammatory adipokines and the modulation of cytokine effects [11]. The interaction between reduced muscle quality and enhanced adiposity increases health risks, particularly in older populations [12,13,14,15,16].

The impact of sarcopenia and obesity on oral health is mediated via distinct pathways. A notable association exists between sarcopenia and oral function, primarily attributed to the interaction between muscle mass and masticatory abilities [17,18]. Sarcopenia typically results in reduced masticatory muscle strength, leading to compromised food intake and nutritional deficiencies. Moreover, older individuals with sarcopenia exhibit an increased susceptibility to oral health complications, including periodontal disease and dental caries, owing to difficulties in maintaining adequate oral hygiene [19].

Obesity is strongly associated with chronic systemic inflammation, exacerbating the progression of periodontal disease [20,21]. In addition, individuals with obesity have a higher propensity for recurrent and severe periodontal diseases. This phenomenon is partially attributed to decreased salivary production [22], which compromises the natural cleansing mechanisms of the oral cavity and increases the risk of dental caries [23,24].

Although the individual effects of sarcopenia and obesity on oral health have been previously studied, the combined impact of sarcopenic obesity on oral health remains elusive. Given the established associations among sarcopenia, obesity, and oral health, examining the long-term implications of sarcopenic obesity on oral health outcomes is critical. However, longitudinal studies of sarcopenic obesity, its progression over time, and its relationship with oral health are limited. Considering that both sarcopenia and obesity contribute to physical limitations and adverse health outcomes that can affect oral well-being, it is reasonable to expect that the progression of sarcopenic obesity, which combines these two conditions, is linked to oral health deterioration.

In the cohort study, we aimed to investigate the longitudinal association between sarcopenic obesity and oral health. We hypothesized that oral function is independently associated with physical condition and function, contributing to the development of sarcopenic obesity.

## 2. Material and Methods

This study encompassed independent older adults (aged ≥65 years) from Sasayama, rural Tamba-Sasayama City, Hyogo Prefecture, Japan, who participated in academic studies between June 2016 and December 2023. This cohort was designated the Frail Elderly in the Tamba-Sasayama Area (FESTA) study. The study was conducted in accordance with the ethical standards established by the Ethics Committees of both Hyogo Medical University (approval number: Rinhi-0342) and Niigata University (Approval number: G2021-0027).

The recruitment process was executed by placing newspaper advertisements and posters at the Sasayama Medical Center and Hyogo Medical University, resulting in the voluntary participation of study subjects. To be eligible for participation, individuals were required to meet the following criteria: they had to be independent older adults aged ≥65 years, residing in the Tamba-Sasayama region of Hyogo Prefecture, capable of traveling to the Sasayama Medical Center via public transportation or private vehicle, and without cognitive impairment (MMSE score >22). From among 1016 participants, 597 surveyed at least twice at a minimum interval of two years were included in the analysis. Individuals who could not ambulate independently (except those using a cane) were excluded. Participants were provided with comprehensive information regarding the objectives and methodology of the study, and written informed consent was obtained before their participation.

### 2.1. Evaluation Items

Participants were required to provide information regarding their medical and smoking histories via a questionnaire. Blood pressure was measured using a fully automatic calibrated oscillator (BP-203 RVII, Colin Co., Kyoto City, Japan).

Body composition was assessed via bioelectrical impedance analysis (BIA) using an InBody 770 device (InBody Japan, Inc., Koto Ward, Tokyo, 136-0071, Japan). Body mass index (BMI), limb skeletal muscle mass (LBM), and percent body were determined using BIA (InBody Co., Seoul, Republic of Korea).

### 2.2. Diagnosis of Sarcopenic Obesity

**Figure 1:**
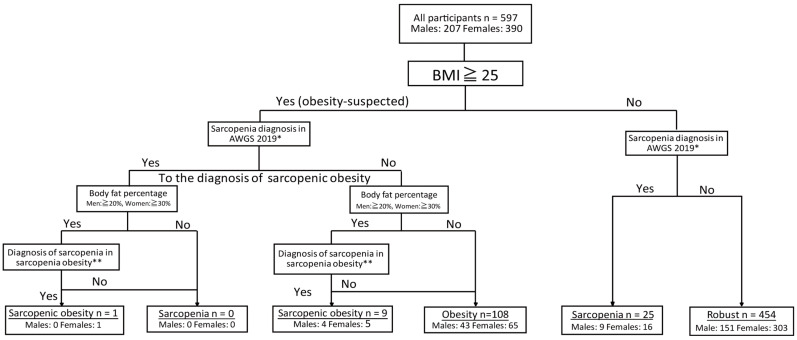
Classification of participants with sarcopenic obesity. *: Low skeletal muscle mass (kg/m^2^) + low muscle strength or/and low physical function (diagnosis of sarcopenia [25]). **: Low skeletal muscle mass (kg/BMI) + low muscle strength or low physical function (diagnosis of sarcopenia obesity [26].

The participants were categorized into four groups based on their health status: robust, obese, sarcopenic, and sarcopenic obese. The classification method follows the flowchart depicted in Figure 1.

First, obesity screening was conducted according to the criteria established by the Japan Society for the Study of Obesity [26]. Individuals with a BMI of ≥25 kg/m^2^ were classified as “obesity-suspected”. Sarcopenic obesity was diagnosed based on the criteria established by the Japanese Society of Gerontology [26], in which obesity was determined by body fat percentage with thresholds set at ˃20% for males and ˃30% for females.

Sarcopenia was defined using the same diagnostic criteria as for sarcopenia alone, incorporating skeletal muscle mass, grip strength, and five-times-sit-to-stand-test (FTSST) performance. However, in the case of sarcopenic obesity, muscle mass was adjusted for BMI, and values of <0.789 for males and <0.512 for females were deemed to indicate low muscle mass [26]. Sarcopenic obesity was diagnosed when an individual met the criteria for both obesity and sarcopenia, as described above.

Sarcopenia was diagnosed based on the criteria set by the Asian Working Group for Sarcopenia (AWGS) [25], incorporating assessments of muscle strength (grip strength), physical function (FTSST performance), and muscle mass (height-adjusted appendicular skeletal muscle mass in kg/m^2^). Sarcopenia was identified in individuals who exhibited both reduced muscle strength and physical function, as well as low muscle mass. Muscle weakness was defined as grip strength of <28 kg in males and <18 kg in females, while a decline in physical function was indicated by an FTSST completion time exceeding 12 s [25]. Muscle mass was assessed using the skeletal muscle mass index (SMI), with values below 7.0 kg/m^2^ in males and 5.7 kg/m^2^ in females classified as having low muscle mass [25].

Based on these criteria, individuals with low muscle mass, reduced muscle strength, and physical function were diagnosed with severe sarcopenia. Those with low muscle mass and either reduced muscle strength or reduced physical function were diagnosed with “sarcopenia”. Both groups were jointly categorized as the sarcopenia group. Participants who did not meet the criteria for either obesity-suspected or sarcopenia were classified as the robust group.

In addition, sarcopenic obesity was further categorized into two distinct stages based on the disease severity. Stage I sarcopenic obesity was characterized by low muscle strength, reduced physical function, low muscle mass, and obesity. Stage II sarcopenic obesity was defined by a further decline in muscle strength and physical function, low muscle mass, obesity, and the presence of comorbidities. Comorbidities were defined as the presence of at least one chronic disease in individuals aged ≥70 years, including metabolic disorders, liver disease, kidney disease, heart disease, respiratory disease, gastric ulcer, osteoporosis, rheumatoid arthritis, thyroid disease, collagen diseases, or stroke, which are all associated with an increased likelihood of sarcopenic obesity [7,26].

### 2.3. Evaluation of Oral Function

The oral function was objectively evaluated by assessing the number of remaining teeth, tongue pressure, occlusal force, masticatory performance, and oral diadochokinesis (ODK). A comprehensive oral health assessment was performed by dental professionals who had received more than two hours of training and calibration prior to the survey. The examination was conducted under optimal lighting conditions, with the subject seated in a reclining chair. Participants who routinely wore dentures were assessed while wearing the dentures.

The number of remaining teeth was defined by third molars and roots, and implants, bridges, and dentures were excluded. Tongue pressure was measured twice using a JMS Tongue Pressure Measuring Device (JMS Co., Ltd., Hiroshima, Japan), and the highest value was recorded [27]. The occlusal force was quantified using an Occlusal Force Meter (GM10, Nagano Keiki, Tokyo, Japan) [28,29]. The maximum occlusal forces on the left and right sides were measured, and their sum was used in subsequent analysis [30,31]. Masticatory performance was evaluated using a standardized masticatory performance evaluation method (scoring method) using gummy jelly [31]. The participants chewed gummy jelly (UHA Mikakuto, Osaka, Japan) 30 times, followed by a visual evaluation of the expectorated fragments using a 10-level scale ranging from 0 to 9 [32]. Tongue motor function was evaluated using ODK. The articulatory velocity of /ta/ was measured using ODK measurement equipment (KENKOU-KUN Handy; Takei Scientific Instruments Co., Ltd., Niigata, Japan) [30].

### 2.4. Data Analysis

Data are presented as mean ± standard error (SE) for continuous variables. Differences between the two groups were assessed using the Student’s *t*-test. Comparisons among three or more groups were performed using one-way analysis of variance (ANOVA), followed by post hoc multiple comparisons. The Bonferroni correction was applied to adjust the *p*-values for multiple comparisons.

Categorical variables are presented as absolute numbers (*n*) and relative frequencies (%). Differences between categorical variables were analyzed using the chi-square or Fisher’s exact test, as appropriate, based on the expected cell counts.

We conducted Cox regression analysis using the incidence of sarcopenic obesity as the event and the time from baseline to follow-up as the time variable to determine whether oral function was an independent factor contributing to the development of sarcopenic obesity. The independent variable was the onset of sarcopenic obesity, and the explanatory variables were those showing a statistically significant difference between the health status groups in the cross-sectional analysis at baseline. The adjusted variables were sex, age, and health status at baseline (divided into four groups). Those with sarcopenic obesity at baseline were excluded from the analysis. We checked for multicollinearity between the explanatory variables and selected the variable with the highest significance level in cases of overlap. The final model was determined using a stepwise method (variable reduction Wald test) and multiple imputations. The criteria for adding and deleting variables were set at *p* < 0.05 and *p* < 0.10, respectively, with a maximum of 20 iterations.

For subgroup analyses, we conducted Cox regression analyses with sarcopenia onset as the event, targeting participants assigned to the robust or obese group at baseline, and examined factors contributing to the onset of sarcopenia. We also conducted a Cox regression analysis with obesity onset as the event, targeting participants in the robust or sarcopenia groups at baseline, and examined factors contributing to the onset of obesity. The subgroup analyses excluded participants with sarcopenic obesity at baseline or follow-up. Cox regression analysis of the subgroups was based on analysis of the onset of sarcopenic obesity.

Statistical significance was defined as a *p*-value of 0.05. All the statistical analyses were performed using SPSS, version 25.0.0 (IBM Corp., Armonk, NY, USA).

## 3. Results

Table 1 presents the participants’ characteristics.

This study included 597 participants (207 males and 390 females). The mean age was 72.8 ± 0.2 years, with males being slightly older (73.7 ± 0.4 years) than females (72.3 ± 0.3 years) (*p* = 0.003). Regarding obesity indicators, males had a higher mean BMI (23.2 ± 0.2 kg/m^2^) than females (22.2 ± 0.1 kg/m^2^) (*p* < 0.001). However, females had a higher body fat percentage (29.6 ± 0.3%) than males (23.1 ± 0.4%) (*p* < 0.001). Interestingly, a higher proportion of males (72.0%) had high body fat levels than females (48.7%) (*p* < 0.001).

Regarding sarcopenia markers, males had a higher SMI (0.868 ± 0.008 kg/BMI) than females (0.623 ± 0.005 kg/BMI) (*p* < 0.001). Males also had higher grip strength (35.4 ± 0.4 kg) than females (24.4 ± 0.7 kg) (*p* < 0.001).

At baseline, the distribution of sarcopenia and obesity categories was similar between both sexes, with 76.0% classified as robust, 18.1% as obese, 4.2% as sarcopenic, and 1.6% as sarcopenic obesity.

At follow-up, the mean age increased to 75.3 ± 0.2 years. The prevalence of sarcopenia and sarcopenic obesity showed sex-based differences (*p* = 0.046). Among male participants, 8.6% were classified as sarcopenic (including severe sarcopenia) and 2.9% as sarcopenic obese, compared with 5.4% and 1.0% among female participants, respectively. However, the differences between male and female participants were non-significant.

Table 2 shows the relationships between health conditions (robustness, obesity, sarcopenia, and sarcopenic obesity) and related factors.

Significant age differences were detected between the groups (*p* < 0.001). Participants in the sarcopenia and sarcopenic obesity groups were older than those in the robust and obese groups. The mean age increased across all groups at follow-up, with the largest increase noted in the sarcopenia group (from 76.3 to 79.6 years). There were no significant sex differences between the groups at baseline or follow-up. Body composition metrics showed consistent patterns at baseline and follow-up. BMI and body fat percentage were significantly higher in the obese and sarcopenic obesity groups than in the robust and sarcopenia groups (*p* < 0.001). The SMI (kg/BMI) was the lowest in the sarcopenic obesity group at both the baseline and follow-up. Physical function measurements revealed notable differences. Grip strength was significantly lower in the sarcopenia group at baseline (*p* = 0.012) and follow-up (*p* = 0.001). The FTSST time was significantly longer in the sarcopenia and sarcopenic obesity groups at both baseline and follow-up (*p* < 0.001), with the gap widening at follow-up. The prevalence of hypertension was highest in the sarcopenic obesity group at baseline (70.0%) and follow-up (80.0%).

Participants in the sarcopenic obesity group had significantly fewer remaining teeth at baseline (*p* = 0.001). However, this difference was non-significant at follow-up. The obesity group exhibited the highest tongue pressure at both time points (*p* = 0.001).

Table 3 shows changes in health status between baseline and follow-up across four health states: robust, obesity, sarcopenia, and sarcopenic obesity.

Most participants maintained their baseline health status during follow-up, particularly those classified as “robust” (89.9%) and “obesity” (76.9%). Some participants showed improvements; 18.5% of those with obesity at baseline were reclassified as robust at follow-up, 44.0% of participants with sarcopenia improved to robust, and 60.0% of participants with sarcopenic obesity were reclassified as obese.

Conversely, the health of some participants deteriorated; 4.2% of participants classified as robust became obese, 5.5% developed sarcopenia, and 1.9% of obese participants progressed to sarcopenia. Among participants with sarcopenic obesity at baseline, 10.0% improved and became robust, while 30.0% of participants remained sarcopenic obese at follow-up.

The McNemar–Bowker test (χ^2^ = 10.803, df = 6, *p* = 0.095) indicated no statistically significant changes in the overall distribution of health states between baseline and follow-up. During the follow-up period, most “robust” participants (408, 89.9%) maintained their health status, while 46 participants (10.1%) experienced deterioration. Among those with altered health status at baseline, 32 (22.4%) showed improved robustness.

Table 4 shows the results of examining the factors contributing to the occurrence of sarcopenic obesity based on the results of the prospective longitudinal analysis. After Cox regression analysis, four risk factors remained for the development of sarcopenic obesity. Males had a significantly higher risk of developing sarcopenic obesity than females (hazard ratio [HR] = 20.191, 95% confidence interval [CI]: 3.151–129.366, *p* = 0.002). As BMI increased, the risk of developing sarcopenic obesity also increased significantly (HR = 2.118, 95% CI: 1.554–2.886, *p* < 0.001). Furthermore, an increase in skeletal muscle mass significantly decreased the risk of sarcopenic obesity (HR = 0.661, 95% CI: 0.510–0.857, *p* = 0.002). Among oral functions, elevated tongue pressure slightly reduced the risk of sarcopenic obesity (HR = 0.906, 95% CI: 0.829–0.990, *p* = 0.028). The goodness of fit of the model was significant (-2 log-likelihood = 57.618, *p* < 0.001), and four significant predictors were identified from among the nine original variables.

Following the subclass analysis, three variables remained in the final model as risk factors for sarcopenia development. Males had a significantly higher risk of developing sarcopenia than females (HR = 31.231, 95% CI: 9.660–100.974, *p* < 0.001). Individuals exhibiting declining physical function (decline in grip strength or extension of the time to stand from a chair) had a significantly lower risk of developing sarcopenia (hazard ratio [HR] = 0.188, 95% CI: 0.065–0.543, *p* = 0.002). Furthermore, enhanced skeletal muscle mass significantly reduced the risk of developing sarcopenia (HR = 0.571, 95% CI: 0.469–0.695, *p* < 0.001). The goodness of fit of the model was significant (−2 log-likelihood = 286.929, *p* < 0.001).

The final model retained the two variables as risk factors for developing obesity. Males tended to have a higher risk of developing obesity than females, although the difference was non-significant (HR = 2.702, 95% CI: 0.830–8.801, *p* = 0.099). Elevated body fat percentage significantly increased the risk of developing obesity (hazard ratio [HR] = 1.192, 95% CI: 1.095–1.297, *p* < 0.001). These results were obtained using a stepwise reduction of variables (Wald test). The goodness of fit of the model was significant (−2 log-likelihood = 187.068, *p* < 0.001). Oral function variables were non-significant explanatory variables in the development of sarcopenia or obesity.

## 4. Discussion

In the current study, we aimed to elucidate the longitudinal relationship between sarcopenic obesity and oral function in independent older adults. Sex, body composition, and muscle mass were the most consistent predictors across outcomes. Additionally, tongue pressure was associated with the risk of sarcopenic obesity but was not a significant predictor of sarcopenia or obesity.

### 4.1. Characteristics of Sarcopenic Obesity

We showed that individuals with sarcopenic obesity were significantly older, exhibited higher body fat, and had poorer muscle function (longer FTSST times) than their robust or obese counterparts. These findings reinforce the notion that sarcopenic obesity is a more severe phenotype resulting from the interplay between sarcopenia and obesity. Furthermore, the high prevalence of comorbid conditions in this group, such as diabetes and hypertension, aligns with previously reported findings [33,34], underscoring the systemic impact of sarcopenic obesity on metabolic and cardiovascular health.

Prospective analyses revealed that males have a substantially higher risk of developing sarcopenic obesity than females. This may be due to sex-based differences in body composition [35], hormonal changes [36], lifestyle factors [37], or oral health behaviors [37]. These findings highlight the need for sex-specific interventions to mitigate sarcopenic obesity and its associated health risks.

For gender differences in sarcopenic obesity, previous studies have shown that aging in men is associated with decreased testosterone secretion, which induces skeletal muscle loss [38]. Reduced testosterone levels are also linked to increased visceral fat accumulation, as decreased physical activity and energy imbalance result in unused energy being stored as visceral fat. Furthermore, gender differences in dietary habits may contribute to the higher risk of sarcopenic obesity in men. Visceral fat secretes inflammatory cytokines, which may play a significant role in the development of sarcopenic obesity [6,21]. These sex-related differences may explain the higher hazard ratio observed for males in this study.

In addition, the prevalence of sarcopenic obesity revealed a slight decline over time, with some cases showing improvement or reclassification into obesity or robust categories, respectively. These trends suggest that targeted interventions focusing on weight management, physical activity, and oral health could reverse or stabilize the progression of sarcopenic obesity in older adults. Exercise interventions, such as resistance training and aerobic exercise, can effectively improve sarcopenic obesity [39]. Furthermore, while the relationship between sarcopenia, malnutrition, and oral frailty has been reported [40,41], reports have also suggested that declining masticatory function can hinder the consumption of meat and other foods high in protein, leading to a decrease in muscle mass [42]; hence, maintaining oral function may be effective in improving sarcopenic obesity.

### 4.2. Associations Between the Incidence of Sarcopenic Obesity and Oral Health

In this study, we confirmed the association between sarcopenic obesity and its development and oral function indices, such as tongue pressure and skeletal muscle mass. Accordingly, a decline in oral function may be associated with the development of sarcopenic obesity; however, the direction of causality remains unclear.

Inadequate nutrition could cause further muscle loss and metabolic abnormalities [43,44]. Specifically, subjects with sarcopenic obesity exhibited the lowest occlusal force and tongue pressure, which are important for chewing and overall oral health, and it was clear that occlusal force had some effect on the progression of sarcopenic obesity. Reduced tongue pressure observed in individuals with sarcopenic obesity may be attributed to several factors. Sarcopenia is associated with lower muscle quality, which can extend to the muscles involved in oral function, potentially reducing tongue pressure [45]. Chronic low-grade inflammation associated with obesity may affect muscle function, including mastication [46]. The loss of muscle mass, a well-known characteristic of sarcopenia, combined with increased fat mass in obesity, may negatively impact the strength of oral muscles [47].

The fact that tongue pressure contributes significantly to nutritional intake through its role in swallowing function and its relationship to systemic skeletal muscle strength makes it a more specific indicator than other oral function measures. Sarcopenic obesity in the elderly is characterized by the accumulation of visceral fat due to age-related loss of muscle mass and reduced physical activity. Previous cross-sectional studies have demonstrated a positive correlation between tongue pressure and total body skeletal muscle mass [48,49]. Furthermore, the tongue plays a critical role in transporting food masses during the feeding and swallowing process [50]. Considering that adequate tongue pressure supports normal nutrient intake and is positively associated with general skeletal muscle function and strength, it may be a specific factor in reducing the risk of developing sarcopenic obesity. Additionally, tongue pressure tends to be associated with sarcopenia more strongly than other oral function indicators, such as the number of teeth or swallowing ability [51]. This suggests that interventions focused solely on dental restoration or prosthetics may be insufficient. Instead, enhancing eating and swallowing functions, along with nutritional support, may be necessary to effectively prevent or manage sarcopenic obesity [52,53].

The relationship between sarcopenic obesity and reduced tongue pressure is likely bidirectional. Lower tongue pressure may lead to difficulties in eating, potentially resulting in a reduced intake of protein-rich foods and contributing to further muscle loss [45]. Conversely, the systemic effects of sarcopenic obesity may lead to reduced muscle strength in the oral system [46].

It is important to note that while our study found a significant association between tongue pressure and sarcopenic obesity, other oral function indicators, such as occlusal force and number of remaining teeth, were not identified as significant predictors in our final model. This finding suggests that the relationship between oral function and sarcopenic obesity may be complex and multifaceted, warranting further investigation [46].

### 4.3. Clinical Implications

Our findings suggest that promoting a balanced nutritional intake could potentially be a useful strategy for preventing or managing sarcopenic obesity. However, our study did not include direct nutritional interventions, and this approach remains a potential avenue for future research. It may be valuable in preventing sarcopenic obesity in older adults without reducing swallowing-related oral muscles while supporting the maintenance of skeletal muscle and physical functions and managing obesity. Additionally, continuously evaluating tongue pressure and monitoring longitudinal changes may be beneficial in delaying or reversing the progression of sarcopenic obesity.

### 4.4. Study Limitations

This study has several limitations. A potential limitation is the relatively small number of participants diagnosed with sarcopenic obesity at baseline (n = 10), which may reduce statistical power and affect generalizability. Additionally, the small sample size may increase the risk of false positives and false negatives, making it difficult to detect significant associations. Future studies with larger sample sizes and more diverse populations are warranted to confirm our results and provide a more comprehensive understanding of the relationship between oral function and sarcopenic obesity.

Furthermore, the study cohort was derived from a specific rural region in Japan, which may limit its generalizability to other populations with different lifestyles or healthcare access. The relatively short follow-up period may also hinder the detection of long-term associations between oral function and sarcopenic obesity. However, previous studies have shown that even shorter periods (3–6 months) can detect changes in body composition related to sarcopenic obesity, suggesting that our follow-up period of over two years is sufficient for this purpose [6].

Additionally, while we attempted to adjust for some potential confounding factors, such as age, sex, and health status, in our statistical models, residual confounding factors may still be present. Factors such as nutritional intake, medication use, and exercise habits could influence both oral function and the development of sarcopenic obesity. Since this study was conducted in Japan, the findings may not be fully applicable to Western populations or other Asian countries with different dietary patterns, exercise habits, and diagnostic criteria for sarcopenic obesity. To better understand these relationships and enhance generalizability, future research should incorporate detailed dietary assessments, such as food frequency questionnaires or dietary recalls, while also focusing on larger, more diverse populations with extended follow-up periods to address potential confounders and explore causative mechanisms. Furthermore, this study did not assess comprehensive oral health, particularly periodontal status. Severe periodontitis can cause systemic inflammation and exacerbate muscle deterioration associated with sarcopenic obesity. Future studies should include periodontal assessments to clarify the role of inflammation in this relationship.

## 5. Conclusions

Our study showed that sarcopenic obesity was independently associated with oral function, particularly tongue pressure. Although we did not detect a direct link between sarcopenia/obesity alone and oral function, the combination of sarcopenia and obesity appeared to be strongly associated with oral health. These findings suggest that preserving and enhancing tongue muscles, which are crucial for swallowing and other oral functions, could potentially play a role in delaying the development of sarcopenic obesity. However, further research is required to establish causality and explore the complex relationships between oral function, sarcopenia, and obesity.

Furthermore, our findings demonstrated that reduced tongue pressure was significantly associated with a decreased risk of developing sarcopenic obesity (HR = 0.906, 95% CI: 0.829–0.990, *p* = 0.028). These results suggest that tongue pressure assessment could be a valuable tool in routine geriatric evaluations, particularly for identifying individuals at risk of sarcopenic obesity. However, further research is needed to determine its validity as a definitive screening tool.

Future studies should explore intervention approaches combining nutritional guidance, resistance exercise, and tongue pressure training to improve muscle function. Additionally, developing a staging system for early sarcopenia detection and integrating oral health assessments into geriatric care may enhance diagnostic accuracy and health management.

## Figures and Tables

**Table 1:** Baseline and follow-up characteristics of participants.

A Summary of Participant in Baseline											
		Overall (*n* = 597)			Males (*n* = 207)			Females (*n* = 390)			*p*-Value
Age (yr) *		72.8	±	0.2	73.7	±	0.4	72.3	±	0.3	0.003
Duration (day)		947.8	±	15.2	936.3	±	26.5	954.0	±	18.5	0.579
Smoking history *		177 (29.6%)			152 (73.4%)			25 (6.4%)			<0.001
Obesity											
BMI (kg/m^2^) *		22.6	±	0.1	23.2	±	0.2	22.2	±	0.1	<0.001
Body fat (%) *		27.3	±	0.3	23.1	±	0.4	29.6	±	0.3	<0.001
	High body weight	118 (19.8%)			47 (22.7%)			71 (18.2%)			0.189
	High body fat *	339 (56.8%)			149 (72.0%)			190 (48.7%)			<0.001
Sarcopenia											
Skeletal muscle mass index (kg/BMI) *		0.708	±	0.006	0.868	±	0.008	0.623	±	0.005	<0.001
Low skeletal muscle mass *		92 (15.4%)			51 (24.6%)			41 (10.5%)			<0.001
Skeletal muscle mass index (kg/m^2^) *		6.45	±	0.04	7.40	±	0.05	5.98	±	0.03	<0.001
Low skeletal muscle mass		185 (31.0%)			60 (29.0%)			125 (32.1%)			0.441
Grip strength (kg) *		28.2	±	0.5	35.4	±	0.4	24.4	±	0.7	<0.001
Low muscle strength		46 (7.7%)			18 (8.7%)			28 (7.2%)			0.509
Five times sit-to-stand test (s) *		7.2	±	0.1	7.6	±	0.1	7.0	±	0.1	0.002
Low physical function		20 (3.4%)			9 (4.3%)			11 (2.8%)			0.324
Comorbidities											
Metabolic diseases											
	Diabetes mellitus *	61 (10.2%)			34 (16.4%)			27 (6.9%)			<0.001
	Hypertension *	253 (42.2%)			100 (48.3%)			152 (39.0%)			0.028
	Hyperlipemia	140 (23.5%)			40 (19.3%)			100 (25.6%)			0.083
	Cardiovascular *	40 (6.7%)			21 (10.1%)			19 (4.9%)			0.014
Asthma		14 (2.3%)			4 (1.9%)			10 (2.6%)			0.627
Tuberculosis		5 (0.8%)			2 (1.0%)			3 (0.8%)			
Pneumonia		10 (1.7%)			5 (2.4%)			5 (1.3%)			0.304
Blood pressure											
SBP (mmHg) *		139.0	±	0.7	136.7	±	1.2	140.2	±	0.8	0.013
DBP (mmHg) *		80.3	±	0.4	79.1	±	0.7	81.0	±	0.5	0.039
Diagnosis of sarcopenia obesity											
Robust		454 (76.0%)			151 (72.9%)			303 (77.7%)			0.835
Obesity		108 (18.1%)			43 (20.8%)			65 (16.7%)			
Sarcopenia											
Sarcopenia		23 (3.9%)			8 (3.9%)			15 (3.8%)			
Severe sarcopenia		2 (0.3%)			1 (0.5%)			1 (0.5%)			
Sarcopenic obesity											
Stage I		2 (0.3%)			1 (0.5%)			1 (0.3%)			
Stage II		8 (1.3%)			3 (1.4%)			5 (1.3%)			
Oral function											
	Remaining teeth	20.9	±	0.3	20.7	±	0.6	21.0	±	0.4	0.713
	Occlusal force (kg) *	59.5	±	1.4	66.4	±	2.8	55.9	±	1.6	<0.001
	Tongue pressure (kg)	33.5	±	0.3	34.0	±	0.6	33.2	±	0.4	0.295
	Oral diadochokinesis *	30.5	±	0.2	29.2	±	0.5	31.1	±	0.3	<0.001
Kihon checklist											
Masticatory function		105 (17.6%)			29 (14.0%)			76 (19.5%)			0.094
Swallowing function		146 (24.5%)			51 (24.6%)			95 (24.4%)			0.940
Dry mouth		178 (29.8%)			56 (27.1%)			122 (31.3%)			0.282
**A summary of participant in follow-up**											
		**Overall** **(*n* = 597)**			**Males** **(*n* = 207)**			**Females** **(*n* = 390)**			***p*-Value**
Age *		75.3	±	0.2	76.2	±	0.4	74.9	±	0.3	0.006
Smoking history *		177 (29.6%)			152 (73.4%)			25 (6.4%)			<0.001
Obesity											
BMI (kg/m^2^) *		22.5	±	0.1	23.1	±	0.2	22.2	±	0.1	0.001
Body fat (%) *		27.3	±	0.3	23.2	±	0.4	29.6	±	0.4	<0.001
	High body weight	118 (19.8%)			43 (20.8%)			75 (19.2%)			0.652
	High body fat *	330 (55.3%)			143 (69.1%)			187 (47.9%)			<0.001
Sarcopenia											
Skeletal muscle mass index (kg/BMI) *		0.697	±	0.006	0.857	±	0.008	0.612	±	0.005	<0.001
	Low skeletal muscle mass *	110 (18.4%)			54 (26.1%)			56 (14.4%)			<0.001
Skeletal muscle mass index (kg/m^2^) *		6.38	±	0.04	7.30	±	0.05	5.90	±	0.03	<0.001
	Low skeletal muscle mass *	113 (18.9%)			68 (32.9%)			45 (11.5%)			<0.001
Grip strength (kg) *		26.8	±	0.3	34.0	±	0.4	22.9	±	0.2	<0.001
	Low Muscle strength *	62 (10.4%)			29 (14.0%)			33 (8.5%)			0.034
Five times sit-to-stand test (s) *		7.3	±	0.1	7.6	±	0.2	7.2	±	0.1	0.038
	Low Physical function	19 (3.2%)			10 (4.8%)			9 (2.3%)			0.095
Comorbidities											
Metabolic diseases											
	Diabetes mellitus *	71 (11.9%)			35 (16.9%)			36 (9.2%)			0.006
	Hypertension *	261 (43.7%)			102 (49.3%)			159 (40.8%)			0.046
	Hyperlipemia	154 (25.8%)			46 (22.2%)			108 (27.7%)			0.146
	Cardiovascular *	52 (8.7%)			25 (12.1%)			27 (6.9%)			0.034
Asthma		14 (2.3%)			4 (1.9%)			10 (2.3%)			0.627
Tuberculosis		7 (1.2%)			2 (1.0%)			5 (1.3%)			0.733
Pneumonia		15 (2.5%)			8 (3.9%)			7 (1.8%)			0.124
Blood pressure											
SBP (mmHg) *		139.6	±	0.7	137.5	±	1.1	140.7	±	0.9	0.034
DBP (mmHg)		79.6	±	0.5	78.4	±	0.7	80.2	±	0.6	0.064
Diagnosis of sarcopenia obesity *											
Robust		440 (73.7%)			146 (70.5%)			294 (75.4%)			0.046
Obesity		108 (18.1%)			37 (17.9%)			71 (18.2%)			
Sarcopenia											
Sarcopenia		36 (6.0%)			15 (7.2%)			21 (5.4%)			
Severe sarcopenia		3 (0.5%)			3 (1.4%)			0 (0.0%)			
Sarcopenia obesity											
Stage I		0 (0.0%)			0 (0.0%)			0 (0.0%)			
Stage II		10 (1.7%)			6 (2.9%)			4 (1.0%)			
Oral function											
	Remaining teeth	20.1	±	0.3	19.9	±	0.6	20.2	±	0.4	0.619
	Occlusal force (kg)	48.9	±	1.4	51.6	±	2.6	47.4	±	1.7	0.153
	Tongue pressure (kg) *	33.0	±	0.4	34.1	±	0.6	32.3	±	0.4	0.015
	Oral diadochokinesis *	30.4	±	0.2	29.4	±	0.4	30.9	±	0.2	0.001
Kihon checklist											
Masticatory function		115 (20.5%)			44 (22.6%)			71 (19.3%)			0.368
Swallowing function		148 (26.3%)			53 (27.2%)			95 (25.9%)			0.740
Dry mouth		176 (31.4%)			52 (26.8%)			124 (33.9%)			0.086

* Data are presented as mean ± SE. *p* < 0.05, calculated using Student’s *t*-test, chi-squared test, or Fisher’s exact test for sex differences. Definitions: duration, number of days from baseline to follow-up. The diagnostic criteria and classification methods for sarcopenic obesity, including BMI, skeletal muscle mass index, muscle strength, and physical function, have been detailed in the ’Diagnosis of Sarcopenic Obesity’ section. Participants were grouped into robust, obese sarcopenia (sarcopenia and severe sarcopenia), and sarcopenia obesity (sarcopenic obesity stages I and II) groups. Abbreviations: SBP, mean systolic blood pressure; DBP, mean diastolic blood pressure. Oral diadochokinesis: Represented tongue motor function by the “ta” sound.

**Table 2:** The relationship between sarcopenic obesity and associated factors.

(a): Baseline		Robust (*n* = 454)			Obesity (*n* = 108)			Sarcopenia (*n* = 25)			Sarcopenic obesity (*n* = 10)			*p*-Value	
Age *		72.5	±	0.3	72.6	±	0.5	76.3	±	1.5	78.3	±	1.2	<0.001	B, C, D, E
Sex															
Male		151 (72.9%)			43 (20.8%)			9 (4.3%)			4 (1.9%)			0.614	
Female		303 (77.7%)			65 (16.7%)			16 (4.1%)			6 (1.5%)				
Smoking history		130 (28.6%)			37 (34.3%)			7 (28.0%)			3 (30.0%)			0.715	
Obesity															
BMI (kg/m^2^) *		21.6	±	0.1	26.5	±	0.1	20.7	±	0.4	28.8	±	0.8	<0.001	A, C, D, E, F
Body fat (%) *		25.5	±	0.3	33.8	±	0.5	27.2	±	1.3	39.2	±	1.2	<0.001	A, C, D, F
Sarcopenia															
	Skeletal muscle mass index (kg/BMI) *	0.726	±	0.007	0.663	±	0.014	0.623	±	0.028	0.546	±	0.040	<0.001	A, B, C
	Skeletal muscle mass index (kg/m^2^) *	6.33	±	0.04	7.09	±	0.08	5.56	±	0.15	6.97	±	0.32	<0.001	A, B, D, F
Grip strength (kg) *		28.5	±	0.7	29.5	±	0.9	20.8	±	1.1	22.4	±	2.9	0.012	B, D
	Five times sit-to-stand test (s) *	7.0	±	0.1	7.4	±	0.2	9.3	±	0.7	9.4	±	1.1	<0.001	B, C, D, E
Comorbidities															
Metabolic diseases															
Diabetes mellitus		45 (9.9%)			10 (9.3%)			3 (12.0%)			3 (30.0%)			0.212	
Hypertension *		179 (39.4%)			58 (53.7%)			8 (32.0%)			7 (70.0%)			0.009	
Hyperlipemia		112 (24.7%)			23 (21.3%)			5 (20.0%)			0 (0.0%)			0.274	
Cardiovascular diseases		29 (6.4%)			9 (8.3%)			2 (8.0%)			0 (0.0%)			0.725	
Respiratory diseases															
Asthma		11 (2.4%)			3 (2.8%)			0 (0.0%)			0 (0.0%)			0.816	
Tuberculosis		4 (0.9%)			0 (0.0%)			1 (4.0%)			0 (0.0%)			0.260	
Pneumonia		8 (1.8%)			1 (0.9%)			1 (4.0%)			0 (0.0%)			0.710	
Blood pressure															
SBP (mmHg) *		137.9	±	0.8	142.3	±	1.5	139.3	±	3.6	151.3	±	5.2	0.009	
DBP (mmHg) *		79.7	±	0.5	83.1	±	0.9	76.6	±	2.4	87.2	±	2.7	0.001	A, D, F
Oral function															
	Remaining teeth *	21.3	±	0.4	20.2	±	0.8	20.2	±	1.9	10.8	±	3.2	0.001	C, E, F
	Occlusal force (kg) *	60.3	±	1.6	60.2	±	3.8	54.4	±	6.7	30.0	±	6.0	0.042	C, E
	Tongue pressure (kg) *	33.0	±	0.4	36.8	±	0.8	29.2	±	1.3	32.5	±	3.6	<0.001	A, D
	Oral diadochokinesis *	30.9	±	0.3	30.3	±	0.5	28.2	±	1.1	24.6	±	2.0	0.001	C, E
Kihon checklist															
Masticatory function		78 (17.2%)			20 (18.5%)			6 (24.0%)			1 (10.0%)			0.748	
Swallowing function *		109 (24.0%)			21 (19.4%)			11 (44.0%)			5 (50.0%)			0.017	
Dry mouth		136 (30.0%)			30 (27.8%)			10 (40.0%)			2 (20.0%)			0.590	
**(b): Follow-up**		**Robust (*n* = 440)**			**Obesity** **(*n* = 108** **)**			**Sarcopenia** **(*n* = 39)**			**Sarcopenic obesity (*n* = 10)**			***p*-Value**	
Age *		74.9	±	0.3	75.1	±	0.5	79.6	±	1.1	80.3	±	1.5	<0.001	B, C, D, E
Sex															
Male		146 (70.5%)			37 (17.9%)			18 (8.7%)			6 (2.9%)			0.136	
Female		294 (75.4%)			71 (18.2%)			21 (5.4%)			4 (1.0%)				
Smoking history		125 (28.4%)			34 (31.5%)			15 (38.5%)			3 (30.0%)			0.583	
Obesity															
BMI (kg/m^2^) *		21.6	±	0.1	26.7	±	0.2	20.7	±	0.4	27.0	±	0.5	<0.001	A, B, C, D, F
Body fat (%) *		25.5	±	0.3	35.0	±	0.5	24.6	±	1.1	37.0	±	1.5	<0.001	A, C, D, F
Sarcopenia															
Skeletal muscle mass index (kg/BMI) *		0.718	±	0.007	0.633	±	0.013	0.671	±	0.024	0.585	±	0.042	<0.001	A, C
Skeletal muscle mass index (kg/m^2^) *		6.28	±	0.04	6.97	±	0.09	5.82	±	0.12	6.75	±	0.34	<0.001	A, B, D, F
Grip strength (kg) *		27.1	±	0.3	27.9	±	0.8	20.3	±	0.8	24.7	±	2.7	<0.001	B, D
Five times sit-to-stand test (s) *		7.1	±	0.1	7.2	±	0.2	8.9	±	0.6	11.9	±	1.0	<0.001	B, C, D, E, F
Comorbidities															
Metabolic diseases															
Diabetes mellitus		53 (12.0%)			12 (11.1%)			4 (10.3%)			3 (30.0%)			0.355	
Hypertension *		184 (41.8%)			63 (58.3%)			16 (41.0%)			8 (80.0%)			0.002	
Hyperlipemia		117 (26.6%)			30 (27.8%)			8 (20.5%)			2 (20.0%)			0.797	
Cardiovascular diseases		44 (10.0%)			10 (9.3%)			2 (5.1%)			0 (0.0%)			0.559	
Respiratory diseases															
Asthma		10 (2.3%)			5 (4.6%)			1 (2.6%)			0 (0.0%)			0.546	
Tuberculosis		5 (1.1%)			0 (0.0%)			2 (5.1%)			0 (0.0%)			0.083	
Pneumonia		13 (3.0%)			2 (1.9%)			2 (5.1%)			0 (0.0%)			0.698	
Blood pressure															
SBP (mmHg)		138.3	±	0.8	142.2	±	1.4	145.1	±	3.3	146.0	±	4.5	0.015	
DBP (mmHg)		79.2	±	0.5	81.0	±	0.9	81.4	±	2.3	75.0	±	1.7	0.158	
Oral function															
Remaining teeth		20.5	±	0.4	19.4	±	0.8	18.8	±	1.6	17.0	±	3.1	0.290	
Occlusal force (kg)		49.3	±	1.6	51.7	±	3.6	41.2	±	4.4	25.5	±	4.0	0.079	
Tongue pressure max (kg) *		32.4	±	0.4	36.2	±	0.8	31.0	±	1.5	29.1	±	4.1	<0.001	A, D
Diadochokinesis		30.5	±	0.2	30.2	±	0.5	29.7	±	0.9	26.8	±	2.2	0.124	
Kihon checklist															
Masticatory function		85 (20.6%)			19 (18.4%)			10 (26.3%)			1 (11.1%)			0.671	
Swallowing function *		110 (26.7%)			19 (18.4%)			15 (39.5%)			4 (44.4%)			0.041	
Dry mouth *		134 (32.7%)			21 (20.4%)			19 (50.0%)			2 (22.2%)			0.006	

Table 2a shows the baseline, and 2b shows the follow-up. Data are presented as mean ± SE. * indicates a significant difference according to one-way analysis of variance, Pearson’s chi-square test, or Fisher’s exact test. The *p*-value was calculated using the one-way analysis of variance or Pearson’s chi-square test/Fisher’s Exact Test, or calculated using Pearson’s chi-square and Mann-Whitney U tests, with Bonferroni correction applied for multiple comparisons. The significance level was set at 5%. Significant differences between groups are denoted as follows: A, a significant difference exists between the robust and obesity groups; B, a significant difference exists between the robust and sarcopenia groups; C, a significant difference exists between the robust and sarcopenic obesity groups; D, a significant difference exists between the obesity and sarcopenia groups; E, a significant difference exists between the obesity and sarcopenic obesity groups; F, a significant difference exists between the sarcopenia and sarcopenic obesity groups. The variable descriptions are the same as in Table 1.

**Table 3:** Changes in health status between baseline and follow-up.

		Follow-Up			
		Robust (440)	Obesity (108)	Sarcopenia (39)	Sarcopenic Obesity (10)
**Baseline**	**Robust (454)**	408 (89.9%)	19 (4.2%) A	25 (5.5%) B	2 (0.4%) †
	**Obesity (108)**	20 (18.5%)	83 (76.9%)	2 (1.9%) B	3 (2.8%) †
	**Sarcopenia (25)**	11 (44.0%)	-	12 (48.0%)	2 (8.0%) †
	**Sarcopenic obesity (10)**	1 (10.0%) *	6 (60.0%) *	-	3 (30.0%) *

Percentages indicate the proportion of participants within each baseline or follow-up group. A: events in the Cox regression analysis of the onset of obesity. B: events in the Cox regression analysis of the onset of sarcopenia. †: group with onset of sarcopenic obesity (events in the Cox regression analysis of sarcopenic obesity). * excluded from all Cox regression analyses.

**Table 4:** Factors contributing to the occurrence of sarcopenic obesity, sarcopenia, and obesity.

	B	Standard Error of B	Wald	*p*-Value	Exp(B)	95.0% CI for Exp(B)	
						Lower	Upper
**Model 1: Development of sarcopenic obesity**							
Sex (Males = 1; Females = 0)	3.005	0.948	10.056	0.002	20.191	3.151	129.366
BMI	0.750	0.158	22.553	<0.001	2.118	1.554	2.886
Tongue pressure	−0.099	0.045	4.799	0.028	0.906	0.829	0.990
Limb skeletal muscle mass	−0.414	0.132	9.813	0.002	0.661	0.510	0.857
**Model 2: Development of sarcopenia**							
Sex (Males = 1; Females = 0)	3.441	0.599	33.041	<0.001	31.231	9.660	100.974
Either decreased grip strength or prolonged chair time	−1.672	0.542	9.521	0.002	0.188	0.065	0.543
Limb skeletal muscle mass	−0.561	0.100	31.275	<0.001	0.571	0.469	0.695
**Model 3: Development of obesity**							
Sex (Males = 1; Females = 0)	0.994	0.602	2.723	0.099	2.702	0.830	8.801
Body fat	0.176	0.043	16.546	<0.001	1.192	1.095	1.297

Cox regression analysis (stepwise variable reduction method (Wald)). The criteria for adding and removing variables were set at *p* < 0.05 and *p* < 0.10, respectively, with a maximum of 20 iterations. B: partial regression coefficient, Exp (B): hazard ratio. Events: incidence of sarcopenic obesity (model 1), sarcopenia (model 2), and obesity (model 3). Time variable: number of days from baseline to follow-up. The time from baseline to follow-up was used as a variable to determine whether oral function was an independent factor contributing to the development of sarcopenic obesity. Explanatory variables: model 1, age, sex, baseline health status (robust, obese, sarcopenic), body mass index (BMI), limb skeletal muscle mass, decreased grip strength or increased chair time, hypertension, oral diadochokinesis (ODK), and chewing performance; model 2, age, sex, baseline health status (robust, obese), BMI, limb skeletal muscle mass, loss of grip strength or prolonged chair time, hypertension, tongue pressure, and chewing performance; model 3, age, sex, baseline health status (robust or sarcopenic), BMI, limb skeletal muscle mass, body fat, hypertension, tongue pressure, and chewing performance.

## Data Availability

Data supporting the findings of this study are available from the corresponding author upon reasonable request. However, the data are not publicly available due to privacy and ethical restrictions.
